# A pilot study: Comparing a novel noninvasive measure of cerebrovascular stability index with an invasive measure of cerebral autoregulation in neonates with congenital heart disease

**DOI:** 10.1017/cts.2023.581

**Published:** 2023-07-10

**Authors:** Carlin A. Merkel, Kenneth M. Brady, Jodie K. Votava-Smith, Nhu N. Tran

**Affiliations:** 1 Keck School of Medicine, Los Angeles, CA, USA; 2 Division of Cardiac Anesthesia, Northwestern University Feinberg School of Medicine and Ann & Robert H, Lurie Children’s Hospital of Chicago, Chicago, IL, USA; 3 Division of Cardiology, Department of Pediatrics, Children’s Hospital Los Angeles, Los Angeles, CA, USA; 4 Division of Neonatology, Department of Pediatrics, Fetal and Neonatal Institute, Children’s Hospital Los Angeles, Los Angeles, CA, USA

**Keywords:** Cerebral autoregulation, congenital heart disease, neonates, fractional tissue oxygen extraction, cerebrovascular stability index

## Abstract

Infants with congenital heart disease (CHD) may have impaired cerebral autoregulation (CA) associated with cerebral fractional tissue oxygen extraction (FTOE). We conducted a pilot study in nine CHD neonates to validate a noninvasive CA measure, cerebrovascular stability index (CSI), by eliciting responses to postural tilts. We compared CSI to an invasive measure of CA and to FTOE collected during tilts (FTOE_Spot_). FTOE_Spot_ correlated with CSI, as did the change in FTOE during tilts, but CSI’s correlation with impaired CA did not reach significance. Larger trials are indicated to validate CSI, allowing for noninvasive CA measurements and measurements in outpatient settings.

## Introduction

Infants with congenital heart disease (CHD) have poorer neurodevelopmental outcomes compared to their healthy counterparts [1,2]. This may result from impaired cerebral autoregulation (CA) or the inability to adequately regulate cerebral blood flow with changes in cerebral perfusion pressure. Previous studies associated impaired CA with greater cerebral fractional tissue oxygen extraction (FTOE), a measure demonstrating cerebral oxygen consumption, in CHD infants [2–4]. This finding suggests increased cerebral metabolic demand may exceed autoregulation capacity. Therefore, elevated FTOE and impaired CA may give insight into cerebral circulation and metabolism in these infants. Examining both may help identify CHD infants at higher risk for altered cerebral blood flow and oxygenation, and potentially, those at risk for adverse neurodevelopmental outcomes.

This study explores a novel noninvasive measure of CA, cerebrovascular stability index (CSI), as a proxy for an invasive method. CSI uses cerebral near infrared spectroscopy (NIRS), a device that measures cerebral oxygenation, to measure regional cerebral oxygen saturation, (rcSO_2_), during postural tilts in which CHD infants are moved from a supine to sitting posture. We calculated CSI as an average change in rcSO_2_ over the postural tilts.

Previous studies characterized CA in both premature and CHD infants, yet none have validated a noninvasive technique of measuring CA that can be utilized outpatient nor in high-risk infants without invasive arterial lines [2,5–7]. The more established method of measuring CA requires a continuous measure of mean arterial pressure (MAP) through an invasive indwelling arterial line [2,5–7]. However, the use of arterial lines is not feasible for clinically stable CHD infants nor healthy infants.

This study aimed to address this clinical gap by validating a noninvasive method of measuring CA (i.e., CSI). We hypothesized that our novel CSI measure would correlate with an invasive CA measure, and that FTOE calculated during spot collection of CSI data, (FTOE_Spot_), would correlate with CSI. Additionally, we investigated an exploratory hypothesis that FTOE would increase from baseline during postural tilts. Validating CSI will promote early identification of impaired CA in CHD infants and those at risk of poor neurodevelopmental outcomes by enabling CA to be measured regardless of clinical status or hospitalization.

## Materials and methods

### Study design

We conducted a pilot study on nine CHD neonates born between June 2018 and April 2019, as a subset of a larger ongoing study examining associations between CA and neurodevelopmental outcomes in CHD infants [8]. This subset included neonates who had two types of hemodynamic data obtained, one using postural tilts to measure CSI and one using clinically indicated arterial lines to measure continuous pre-operative hemodynamics.

### Study sample

We recruited mothers of fetuses with prenatal CHD diagnoses from the Fetal Cardiology Clinic and neonates with CHD diagnosed postnatally from the Cardiothoracic Intensive Care Unit at Children’s Hospital Los Angeles.

We included neonates who were: ≥37 weeks gestational age at birth, diagnosed with CHD either prenatally or postnatally, and had an invasive indwelling arterial line (an inclusion criterion not required for our overarching study). We excluded any neonates who: had hemodynamic instability, were intubated, had additional congenital abnormalities, or were > 14 days old, to allow for postural tilt measures and to eliminate confounding variables.

### Data collection

We collected continuous hemodynamic data pre-operatively for up to 72 hours, which included MAP, measured via pressure transducer connected to an indwelling arterial catheter, pre-ductal systemic arterial saturation (SpO_2_), measured via pulse oximeter, and rcSO_2_ via cerebral NIRS, recorded using an INVOS™ cerebral oximetry infant-neonatal sensor (Covidien, Medtronic, Minneapolis, MN).

We collected CSI using a postural tilt method in which neonates were manually moved from a supine (0°) to sitting posture (90°) with one hand placed on the infants’ back and another supporting the neck [8]. We collected rcSO_2_ values at 5-second intervals for 35 minutes. Baseline rcSO_2_ values were collected for each neonate for 15 minutes while supine. We collected CSI data as the following: (1) 5 minutes of rcSO_2_ in a supine position, (2) 5 minutes of rcSO_2_ in a sitting position, and (3) 2 minutes of rcSO_2_ for two subsequent tilts in both supine and sitting postures. We calculated average rcSO_2_ values using the last two minutes while supine prior to the tilt and the first two minutes while sitting for all three tilts. We calculated the change score as the average sitting minus average supine rcSO_2_ values. We calculated CSI as the average change score over the three postural tilts.

We calculated cerebral oximetry index (CO_x_) as a moving, linear correlation coefficient between MAP and rcSO_2_ collected using 30-paired 10-second samples over a moving 300-second window via ICM + Cambridge software (Cambridge Enterprises, Cambridge, UK) [6]. Impaired CA was defined as a CO_x_ correlation coefficient > 0.3 and the percentage of time spent with impaired CA was defined as the CA measurement for each infant [5]. We defined our CA measurement based on the definition of impaired CA outlined by Brady *et al.* and given the wide use of CO_x_ as an indicator of cerebral oxygenation in neonatal and pediatric populations [9].

We calculated FTOE using SpO_2_ via pulse oximetry and the equation FTOE = (SpO_2_ – rcSO_2_)/SpO_2_. FTOE was calculated both continuously, as an average over the pre-operative monitoring period, FTOE_Cont_, and as a spot check during the postural tilts, as an average of each 2-minute period that CSI data were collected in the sitting posture, FTOE_Spot_. FTOE_Change_ was calculated as FTOE_Spot_ – FTOE_Cont_.

### Statistical analysis

We used SPSS v28 for analyses. We analyzed CSI, CA, and FTOE variables for normality. Pearson’s correlation analyzed the associations between CA and CSI, and between CSI and FTOE_Spot_. Kendall’s tau tested the associations between FTOE_Cont_ and FTOE_Spot,_ and between CSI and FTOE_Change_.

## Results

### Participants’ characteristics

We collected CA and CSI data on nine CHD neonates. Sixty-six percent of the neonates were female and 100% of the neonates were of Latinx background. The average gestational age at birth was 38.7 weeks with an average birth weight of 3.1 kg. Eighty-eight percent of the neonates had cyanotic CHD and 44% had single ventricle CHD. Sociodemographic information, birth characteristics, average time monitored, and CHD diagnosis type are detailed in Table 1. Individual CHD diagnoses are included in Table 2.

**Table 1.** Sociodemographics, baseline characteristics, and congenital heart disease (CHD) types of participants. Results reported as mean ± **SD or N (%).**

Characteristic	CHD infants *N* = 9
**Sex, %**	
Female	6 (66.7)
**Ethnicity, %**	
Latinx	9 (100.0)
**Gestational age at birth**	
Average ± SD	38.7 ± 0.8
**Birth weight, kg**	
Average ± SD	3.1 ± 0.5
**Birth length, cm**	
Average ± SD	48.6 ± 3.5
**Birth head circumference, cm**	
Average ± SD	34.0 ± 1.3
**Gestational age at the time of exam**	
Average ± SD	38.8 ± 1.0
**Weight at the time of exam, kg**	
Average ± SD	3.2 ± 0.5
**Length at the time of exam, cm**	
Average ± SD	48.1 ± 3.4
**Head circumference at the time of exam, cm**	
Average ± SD	34.1 ± 1.4
**Time monitored for pre-op data, hours**	
Average ± SD	25.9 ± 18.5
**CHD type, %**	
Cyanotic lesions	8 (88.9)
Limited aortic outflow	1 (11.1)
Single ventricle	4 (44.4)

**Table 2.** Participant diagnosis and CA, CSI, FTOE_Cont,_ and FTOE_Spot_ data.

Participant	CHD diagnosis	CA	CSI	FTOE_Cont_	FTOE_Cont_ SD	FTOE_Spot_	FTOE_Spot_ SD	Time monitored for pre-op data (hours)
1	LTGA/CoA	14.13	−5.88	0.25	0.05	0.36	0.06	21
2	DORV/TGA/HRV	18.47	4.55	0.14	0.05	0.06	0.01	18
3	TA	8.38	−5.42	0.24	0.07	0.32	0.02	50
4	HLHS/VSD	12.49	−0.01	0.30	0.03	0.31	0.03	19
5	TGA	11.54	−10.89	0.14	0.06	0.33	0.04	1
6	SV/PA/heterotaxia	5.15	−4.98	0.27	0.07	0.27	0.03	18
7	TAPVR to CS	9.68	−5.45	0.26	0.18	0.37	0.05	62
8	TOF	21.27	−4.57	0.17	0.07	0.18	0.03	24
9	HRV	10.45	−5.24	0.32	0.09	0.33	0.03	20

CA = cerebral autoregulation, CoA = coarctation of aorta, CSI = cerebrovascular stability index, DORV = double-outlet right ventricle, FTOE_Cont_ = fractional tissue oxygen extraction calculated continuously, FTOE_Spot_ = fractional tissue oxygen extraction collected during tilts, HRV = heart rate variability, HLHS = hypoplastic left heart syndrome, TA = truncus arteriosus, SV = single ventricle, PA = pulmonary atresia, TAPVR to CS = total anomalous pulmonary return to coronary sinus, TGA = transposition of great arteries, TOF = tetralogy of Fallot, VSD = ventricular septal defect.

### Statistical analysis findings

**Figure 1.**
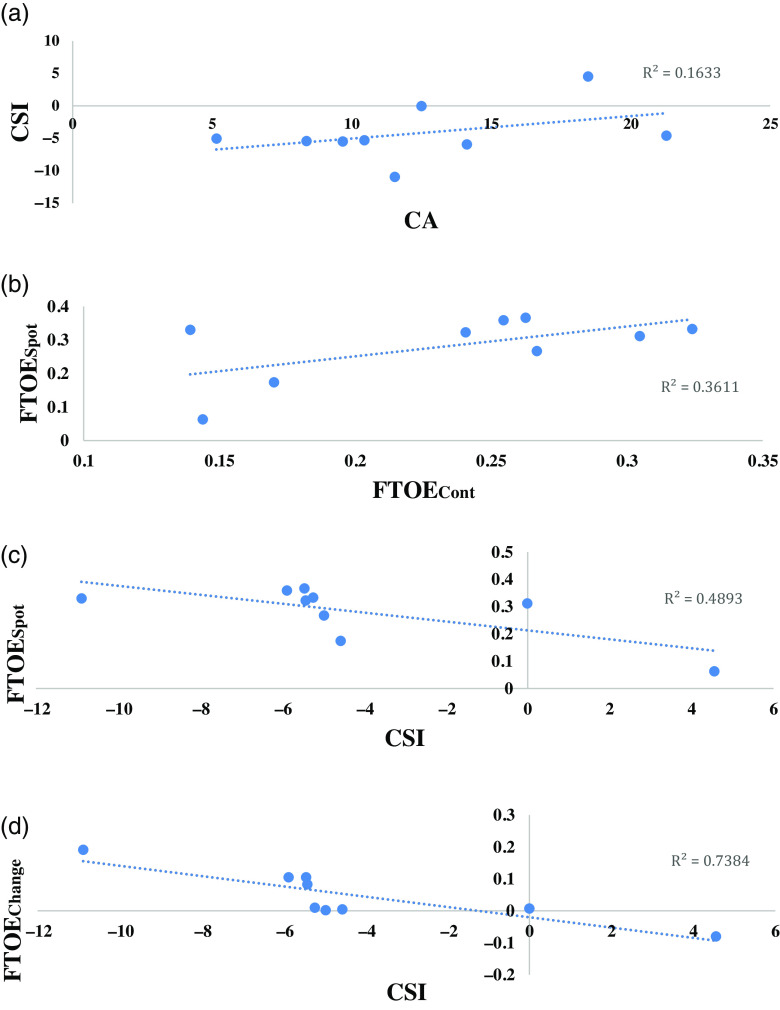
Associations of cerebral autoregulation (CA), cerebrovascular stability index (CSI), and fractional tissue oxygen extraction (FTOE) variables. ***a***: Scatterplot of the association between CA and CSI with a fitted trendline. ***b***: Scatterplot of the association between FTOE_Cont_ and FTOE_Spot_ with a fitted trendline. ***c***: Scatterplot of the association between CSI and FTOE_Spot_ with a fitted trendline. ***d***: Scatterplot of the association between CSI and FTOE_Change_ with a fitted trendline.

CA was moderately associated with CSI, as demonstrated in Fig. 1; however, this association did not reach statistical significance (*r* = 0.40, *p* = 0.28). CSI was moderately associated with FTOE_Spot,_ (*r* = –0.61, *p* = 0.022*) and FTOE_Change,_ (*r* = –0.67, *p* = 0.012*), respectively (Fig. 1c, d). FTOE_Cont_ did not significantly correlate with FTOE_Spot_ (Fig. 1b), (*r* = 0.28, *p* = 0.30). Furthermore, FTOE_Spot_ was greater than FTOE_Cont_ for all neonates except one. Individual FTOE values and total time monitored are detailed in Table 2.

## Discussion

The significant associations found between CSI and FTOE_Spot,_ and CSI and FTOE_Change,_ provide promising support for the use of CSI, despite the association between CSI and CA not reaching statistical significance. With validation of our novel CSI measure, CSI may be implemented as a noninvasive CA measure in CHD neonates and can be extended as a tool for use in other vulnerable populations. This will allow comparisons of brain health to be made between neonates both with and without CHD and between other susceptible populations. Furthermore, early recognition of impaired CA may help identify individuals at risk for future brain injury or neurodevelopmental delays.

The relationship between postural changes, alterations in blood pressure, and subsequently cerebral blood flow has been elucidated in several studies [10,11]. Kim *et al*., identified that cerebral NIRS can determine precise changes in cerebral blood volume when children undergo a postural change from sitting to standing [10], indicating that the cerebral NIRS system is sensitive to detecting changes in cerebral oxygenation over different positions. Moreover, Petrova and Mehta found that moving preterm infants from supine to sitting (in a car seat) led to a reduction in cerebral oxygenation obtained via cerebral NIRS due to a loss of autoregulation [11]. Thus, impaired CA may limit adequate cerebral perfusion following an orthostatic change.

Therefore, we expected similar findings in CHD neonates and anticipated that our CSI measure would provide a sensitive method of measuring CA, given their cerebral vulnerabilities are comparable to preterm infants [12]. We predicted a negative association between CSI and CA, presuming that for infants with more negative CSI values (i.e., rcSO_2_ declined when moved upright), CA would be more positive (i.e., greater time spent with impaired CA). This would indicate that as neonates lost adequate cerebral oxygenation via CSI, they would concurrently demonstrate impaired CA. However, we found that CA did not significantly associate with CSI. This may be due to our small sample size where outliers have significant effect, or it may indicate that greater cerebral oxygen extraction during a postural tilt may not correlate with one’s baseline risk for impaired CA.

Previous studies investigated using noninvasive fast doppler sonography as a method to assess CA and have compared transcranial dopplers (TCDs) to cerebral NIRS [13,14]. Peeples *et al*., determined that fast doppler assessment of the basal ganglia can reliably measure cerebral blood flow velocity as a proxy for cerebral tissue perfusion and consequently autoregulation in preterm infants [13]. Elting et al., compared macrovascular estimates of CA via TCDs and microvascular estimates of CA via NIRS in healthy adults. They established that estimates of CA via either TCDs or NIRS are similar after correcting for confounders [14]. Thus, we infer that the use of either dopplers or NIRS can be applied to measures of CA. Utilizing either as a noninvasive measure lies at the discretion and expertise of researchers and healthcare providers.

Prior studies established that greater FTOE associated with impaired CA in CHD infants [2–4]. Lynch *et al*. demonstrated increased cerebral oxygen extraction due to an aberrant autoregulation response in CHD infants leads to decreased cerebral oxygenation [4]. Furthermore, CHD infants demonstrated greater FTOE and lower rcSO_2_ when non-sedated, indicating greater difficulty maintaining adequate cerebral perfusion when active [2,3]. Thus, we expected CHD infants to have greater FTOE after being moved to a sitting position as compared to while supine. In our sample, all but one neonate had higher FTOE_Spot_ compared to FTOE_Cont_. We attributed this to a greater proportion of time spent awake and upright during positional changes while FTOE_Spot_ was collected as compared to FTOE_Cont_ which was collected while largely supine. Additionally, we found FTOE_Spot_ and FTOE_Change_ both had significant associations CSI. This suggests that the cerebral saturation decline elicited during postural tilts is related to greater cerebral oxygen extraction, i.e., FTOE_Spot_, resulting in a greater change from baseline, i.e., FTOE_Change_. The lack of correlation between FTOE_Cont_ and FTOE_Spot_ may underlie the lack of association between CSI and CA, as baseline measures may not be indicative of the neonatal response to change provoked by moving to an upright posture.

### Limitations

Several limitations are of note in this study. First, to collect the two types of hemodynamic data, measures were taken in clinically stable, non-intubated neonates with arterial lines. Thus, our sample was limited to a small subset of our overarching study, as only nine CHD neonates fit both the inclusion and exclusion criteria. This lowered the statistical power of detecting significant associations. Second, our sample was recruited from the Los Angeles area and every neonate was of Latinx background, limiting the generalizability of our study. Future studies may utilize a larger and nationally representative sample to gain statistical power and demonstrate a significant association between CSI and CA to validate the use of CSI. Furthermore, replication of CSI in other studies will help establish confidence in this noninvasive technique.

## Conclusions

We found significant associations between CSI and FTOE while infants were in a sitting position, and that cerebral oxygen extraction increases from baseline during a postural tilt in most CHD infants, providing promising evidence for the use of the CSI measure in clinical practice. Although our main hypothesis regarding the association of CSI and the percent time spent with impaired CA was not statistically significant, we provide further evidence that FTOE, impaired CA, and CSI are linked. Our study, however, was restricted by a small sample size. Validation of the CSI method may help improve clinical practice as it would allow for measures of CA to be obtained in healthy infants, in outpatient settings, and in vulnerable infants without arterial lines.
